# Lightweight Multimodal Fusion for Urban Tree Health and Ecosystem Services

**DOI:** 10.3390/s26010007

**Published:** 2025-12-19

**Authors:** Abror Buriboev, Djamshid Sultanov, Ilhom Rahmatullaev, Ozod Yusupov, Erali Eshonqulov, Dilshod Bekmuradov, Nodir Egamberdiev, Andrew Jaeyong Choi

**Affiliations:** 1 Department of AI-Software, Gachon University, Sujeong-Gu, Seongnam-si 13120, Republic of Korea; abror1989@gachon.ac.kr; 2 Department of Infocommunication Engineering, Tashkent University of Information Technologies Named After Muhammad Al-Khwarizmi, Tashkent 100200, Uzbekistan; sultanov@tuit.uz; 3 Digital Technologies and Artificial Intelligence Development Research Institute, Tashkent 100125, Uzbekistan; ilhom9001@gmail.com; 4 Department of Exact Sciences, Kimyo International University in Tashkent, Tashkent 100121, Uzbekistan; 5 Department of Software Engineering, Samarkand State University, Samarkand 140104, Uzbekistan; ozodyusupov@gmail.com; 6 Department of Software Engineering, Samarkand State University Named After Sharof Rashidov, Samarkand 140104, Uzbekistan; eshonkuloverali@samdu.uz; 7 Department of Digital Technologies and AI, Tashkent Institute of Irrigation and Agricultural Mechanization Engineers, National Research University, Tashkent 100000, Uzbekistan; bekmurodov.k1958@gmail.com; 8 Department of Convergence of Digital Technologies, Tashkent University of Information Technologies Named After Muhammad Al-Khwarizmi, Tashkent 100200, Uzbekistan; n.egamberdiyev@tuit.uz

**Keywords:** urban ecology, multimodal fusion, tree health monitoring, image–sensor integration, environmental intelligence, multi-task neural networks, carbon sequestration estimation, oxygen production modeling, edge-efficient AI

## Abstract

Rapid urban expansion has heightened the demand for accurate, scalable, and real-time methods to assess tree health and the provision of ecosystem services. Urban trees are the major contributors to air-quality improvement and climate change mitigation; however, their monitoring is mostly constrained to inherently subjective and inefficient manual inspections. In order to break this barrier, we put forward a lightweight multimodal deep-learning framework that fuses RGB imagery with environmental and biometric sensor data for a combined evaluation of tree-health condition as well as the estimation of the daily oxygen production and CO_2_ absorption. The proposed architecture features an EfficientNet-B0 vision encoder upgraded with Mobile Inverted Bottleneck Convolutions (MBConv) and a squeeze-and-excitation attention mechanism, along with a small multilayer perceptron for sensor processing. A common multimodal representation facilitates a three-task learning set-up, thus allowing simultaneous classification and regression within a single model. Our experiments with a carefully curated dataset of segmented tree images accompanied by synchronized sensor measurements show that our method attains a health-classification accuracy of 92.03% while also lowering the regression error for O_2_ (MAE = 1.28) and CO_2_ (MAE = 1.70) in comparison with unimodal and multimodal baselines. The proposed architecture, with its 5.4 million parameters and an inference latency of 38 ms, can be readily deployed on edge devices and real-time monitoring platforms.

## 1. Introduction

The rapid growth of urban environments has intensified the need for sustainable ecological management, particularly continuous monitoring of tree health and its environmental contributions—oxygen production and carbon dioxide absorption [1]. Urban trees are pivotal for air-quality regulation and biodiversity, yet their condition is routinely challenged by heat, drought, pests, diseases, and anthropogenic stressors [2]. Ensuring accurate and timely assessments is therefore central to urban forestry operations and climate-change mitigation strategies [3]. Conventional monitoring—visual surveys by arborists—remains indispensable but is time-consuming, subjective, and hard to scale across large city networks [4]. Advances in machine learning and deep learning now enable automated analysis of high-volume, heterogeneous data, opening a path to more reliable and scalable tree-health assessment [5]. In this context, integrating visual with sensor-based measurements provides complementary information that improves robustness and predictive accuracy compared with either modality alone [6].

This research accomplishes four major contributions. (i) We present a lightweight multimodal learning framework that fuses RGB imagery with environmental and biometric sensor data to perform tree-health classification and ecosystem-service estimation simultaneously. (ii) We architect an efficiency-focused design around EfficientNet-B0 with MBConv and squeeze-and-excitation channel recalibration, along with a small MLP for sensor processing. (iii) We create a single multi-task learning scheme that localizes the classification and regression objectives through a balanced loss-fusion mechanism. (iv) We carry out a comprehensive assessment on a deliberately collected multimodal dataset, which also includes ablation studies, species-level performance analysis, and latency profiling on both workstation-class and edge-device hardware.

The rest of this manuscript is structured as follows. Section 2 surveys the research on tree-health monitoring, multimodal fusion, and multi-task learning. Section 3 describes the proposed model architecture, the multimodal fusion strategy, and the multi-task loss design. Section 4 provides information about the dataset, the experimental setup, baseline comparisons, and ablation studies. Section 5 recaps the results and presents the future research avenues.

## 2. Related Works

The integration of deep learning into environmental monitoring—particularly for tree-health assessment—has accelerated in recent years [7]. While traditional assessments based on manual inspection remain widespread, they are time-consuming and susceptible to observer bias, motivating automated alternatives that exploit high-resolution imagery and dense sensor networks. Early work relied on classical computer-vision pipelines, using texture and shape descriptors for species identification and disease recognition [8,9]. With the advent of convolutional neural networks (CNNs), accuracy and robustness improved substantially [10]. Architectures such as ResNet50, VGG16, and Inception have been applied to species classification and health assessment from RGB data alone [11]; for example, ResNet50 has been shown to differentiate healthy versus diseased foliage by leveraging cues like color and texture variations [12], and CNN-based systems have achieved high accuracy for early disease detection from leaf images [13]. Despite these gains, image-only models are sensitive to illumination, seasonality, occlusions, and background clutter, which limits generalization in heterogeneous urban scenes. To address these challenges, multimodal fusion strategies combine visual signals with environmental and tree-specific attributes [14,15]. A common design encodes RGB inputs with a CNN while processing sensor vectors with a fully connected network, fusing the embeddings to improve prediction stability and accuracy [16]. Temporal variants augment the sensor pathway with recurrent modules to exploit time-series dynamics alongside imagery, enabling prediction of both health status and physiological variables such as oxygen production and CO_2_ absorption [17]. These systems improve performance but can demand sizable training corpora and substantial compute [18].

Recent architectural advances target better accuracy–efficiency trade-offs [19]. Squeeze-and-excitation (SE) mechanisms adaptively recalibrate channel responses, emphasizing informative features and suppressing noise [20]. The SE block, introduced in [21] and subsequently integrated into efficient backbones like MobileNetV2 and EfficientNet, consistently boosts representational capacity with modest overhead [22], benefiting domains including plant-disease recognition [23]. Multi-task learning (MTL) jointly optimizes related objectives, improving data efficiency and generalization [24]. In tree-health monitoring, MTL enables concurrent optimization of health classification with regression targets such as oxygen-production and CO_2_-absorption estimates [25]. Prior work demonstrates that sharing a backbone while using task-specific heads can surpass single-task baselines on both categorical and continuous outcomes [26]. Building on these insights, we adopt an efficiency-oriented hybrid that fuses an image encoder (EfficientNet-B0) with a sensor-aware MLP under an MTL regime. Our approach advances beyond unimodal or loosely coupled systems by (i) tightly integrating sensor context with visual phenotypes, (ii) leveraging MBConv and SE for compact yet expressive representations, and (iii) deploying specialized heads to reduce negative transfer across tasks. Empirically, the proposed model outperforms image-only baselines and sensor-only variants on both health classification and ecological regression metrics, highlighting the value of effective multimodal fusion in resource-constrained urban-forestry applications.

## 3. Materials and Methods

**Figure 1:**
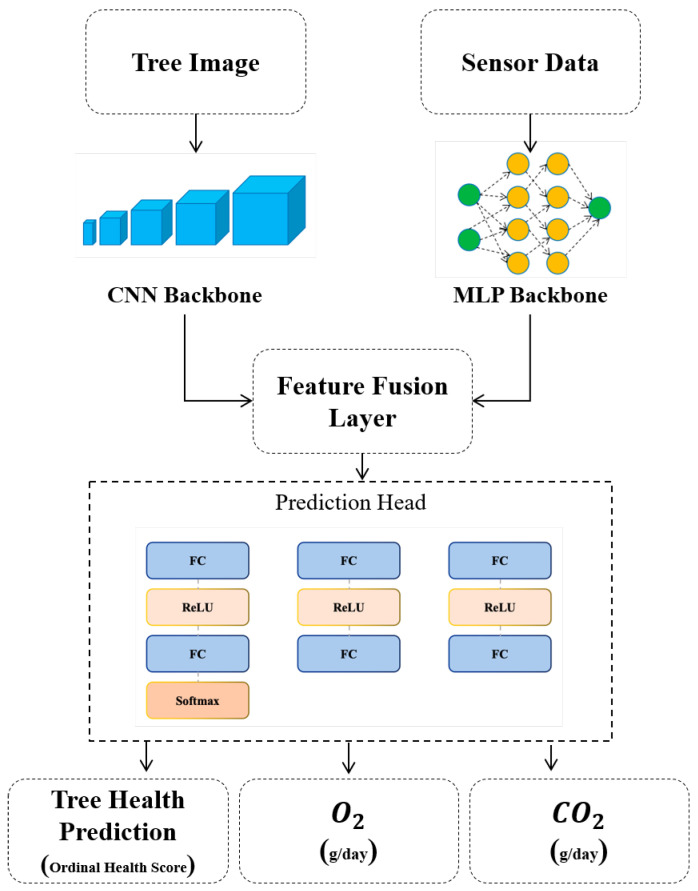
The architecture of the novel proposed model.

In this study, we introduce a novel hybrid architecture that synergistically integrates a deep convolutional feature extractor and a multilayer perceptron to jointly model visual and sensor-based information Figure 1. Specifically, RGB images $X^{W\;x\;H\;x\;C}\in R$ of trees captured for health condition classification are processed through the EfficientNetB0 backbone for hierarchical feature representation, as formulated in Equation (1):

(1) $$F_{{Block}_{1}}=MBConv(F_{3x3}(X))$$

As depicted in Equation (1), the proposed architecture begins by processing the input RGB image through the initial block of the backbone, which serves as the visual feature extractor. The model architecture preserves the standard structure of the Mobile Inverted Bottleneck Convolution (MBConv) block throughout training. Each MBConv unit consistently employs depthwise-separable convolutions for computational efficiency, a squeeze-and-excitation (SE) attention mechanism to enhance channel interdependencies, and a linear projection layer to maintain dimensionality constraints. These components work in concert to extract semantically rich and spatially aware representations from tree images, facilitating accurate classification of tree health status, as shown in Equation (2):

(2) $$MBConv=Concat(Projection(SE(F_{DW}()),Input)$$

The MBConv block initiates with a depthwise separable convolutional operation $F_{DW}$, a computationally efficient alternative to standard convolution. This approach decouples spatial filtering from feature combination, significantly reducing the number of parameters while preserving representational capacity, Equation (3):

(3) $$F_{DW}=\;SiLU(BN(PW(DW())))$$

**Figure 2:**
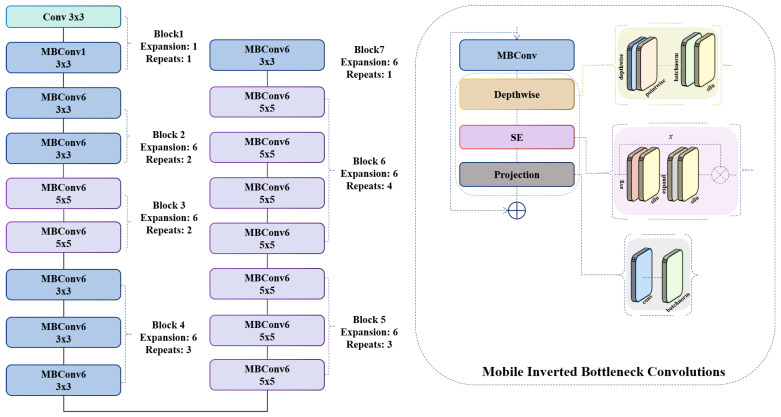
The feature extractor block of the proposed model based on EfficientNetB0.

As illustrated in Equation (4), the internal architecture of the SE block facilitates adaptive channel-wise feature recalibration. Specifically, this mechanism enhances the representational power of the network by explicitly modeling interdependencies between channels. The SE block first squeezes global spatial information into a compact channel descriptor via global average pooling, and then excites the descriptor through a lightweight gating mechanism typically comprising two fully connected layers and a nonlinear activation function such as SiLU before applying the resulting attention weights to scale the original feature maps Figure 2. This selective emphasis on informative channels improves the ability of the system to capture discriminative features while suppressing less relevant ones:

(4) $$SE\;=\;\sigma (W_{2}\delta (W_{1}GAP(F_{DW})))\cdot F_{DW}$$

Equation (5) defines the final projection phase of the MBConv block, where a 1 × 1 pointwise convolution is applied to the output of the SE-enhanced feature map. This operation reduces the number of channels back to the desired output dimensionality, enabling compatibility with subsequent layers. By acting as a bottleneck, the projection layer facilitates efficient feature compression and allows the inclusion of residual connections when input and output dimensions match:

(5) $$Projection\;=\;Concat(BN(F_{1x1}()),\;Input)$$

The sensor data is passed through the MLP, and its output is subsequently concatenated with the feature representations extracted from the input image. This fused vector serves as the unified representation for the downstream prediction heads, Equation (6):

(6) $$F_{fusion\_layer}=\;Concat(Flatten(MLP(X_{sensor}),\;F_{feature\_extr})$$

The fused multi-modal representation is fed into a shared prediction module, which subsequently branches into three distinct task-specific heads.

Figure 2 illustrates the internal structure of the MBConv block used in the EfficientNet-B0 backbone. The operator DW-Conv denotes a depthwise convolution, which applies a spatial convolution independently to each input channel, reducing computational cost while preserving spatial structure. The operator PW-Conv refers to a pointwise 1 × 1 convolution that mixes information across channels. The Expand layer increases the channel dimension by a factor of 4× or 6×, depending on the block configuration, while the Project layer reduces it back to the desired output dimensionality. The SE block performs channel-wise attention: The Squeeze operator applies global average pooling to generate a channel descriptor, and the Excitation operator uses two fully connected layers with a SiLU activation to produce channel-attention weights. These weights re-scale the feature maps via an element-wise multiplication operator. Residual connections are added when the input and output dimensions match, allowing the block to learn efficient transformations without losing original information. Tensor shapes before and after each operator are shown in the figure to clarify how the feature representation evolves throughout the block.

This multi-head architecture facilitates effective multi-task learning by allowing each head to specialize in a specific objective namely, tree health classification, oxygen production estimation, and CO_2_ absorption prediction. Such modularization not only mitigates task interference but also enhances the overall predictive performance and interpretability of the model:

(7) $$F_{{Head}_{1}}=\;Softmax(FC(ReLU(FC(F_{fusion\_layer}))))$$

(8) $$F_{{Head}_{2}}=FC(ReLU(FC(F_{fusion\_layer})))$$

(9) $$F_{{Head}_{3}}=FC(ReLU(FC(F_{fusion\_layer})))$$

Equations (7)–(9) define the output transformations performed by each of the task-specific heads of the proposed model. Each head is designed to independently optimize for a particular target variable namely, health condition classification, oxygen production estimation, and carbon dioxide absorption prediction, respectively. These heads share the same input representation from the fusion layer, yet they are architecturally decoupled to prevent feature entanglement across tasks. This design promotes specialization and stability during training, ensuring that each prediction pathway remains focused on its specific learning objective.

We utilize a weighted sum of the three task-specific losses to train the suggested model under a unified multi-task learning (MTL) framework. The overall objective function is expressed as:

(10) $$L_{T}=\;\lambda_{cls}L_{cls}+\lambda_{O_{2}}L_{O_{2}}+\lambda_{{CO}_{2}}L_{CO_{2}}$$

where $L_{cls}$ denotes the cross-entropy loss used for tree-health classification, and $L_{O_{2}}$ and $L_{CO_{2}}\;$ correspond to the mean absolute error (MAE) losses for oxygen-production and $CO_{2}$-absorption prediction, respectively.

Since the regression goals inherently have bigger numerical scales than the classification loss, all the regression targets are standardized by z-score normalization before the training. This makes sure that the sizes of their gradients stay comparable to those of the cross-entropy classifier. To stop one single task from dominating the joint optimization process, we use a normalized static weighting scheme with coefficients that are tuned empirically as well:

(11) $$\lambda_{cls}=0.4\;\;\;\;\lambda_{O_{2}}=0.3\;\;\;\;\lambda_{{CO}_{2}}=0.3$$

These weights were chosen based on initial experiments that examined loss values and gradient norms for different tasks. We tried different dynamic loss-balancing methods as well, but they led to less stable convergence for the size of our dataset. The ultimate static weighting scheme turned out to be the most stable optimization method, which made it possible for the model to improve both classification performance and regression accuracy to the same extent without giving preference to one of the objectives.

## 4. Experiments and Results

This section evaluates the proposed multimodal, multi-task framework under a unified and reproducible protocol. We first describe the dataset, tasks, and evaluation metrics; then specify baselines and training details; and finally report main results, ablations, and efficiency measurements.

### 4.1. Dataset

In this study, we leverage a custom-curated multimodal dataset tailored for intelligent tree health monitoring in urban environments. The dataset integrates two distinct yet complementary modalities: high-resolution RGB images of trees and structured sensor data reflecting environmental and tree-specific attributes. Each image in the visual dataset corresponds to a unique tree, with efforts made to capture a frontal view under varied lighting and seasonal conditions to ensure visual clarity of the trunk, branches, and canopy. The raw images are resized to a standardized resolution of 224 × 224 pixels to comply with the input requirements of the EfficientNet-B0 backbone. To enhance model robustness and mitigate background noise, tree images undergo background subtraction using semantic segmentation techniques, preserving only the foreground object. Data augmentation techniques such as random cropping, brightness and contrast perturbations, and horizontal flipping are employed to simulate real-world variations and improve generalization.

To figure out how species diversity affected the model’s performance, we looked at the distribution of species in the dataset. There were 12 tree species in total, out of which four species—Platanus orientalis (27%), Acer negundo (18%), Fraxinus pennsylvanica (14%), and Morus alba (12%)—made up 71% of all samples. The remaining eight species together accounted for 2–5% of the dataset each and were considered rare species. As different morphological and physiological characteristics of the species may influence the generalization of vision-based models, we performed an analysis of the performance at the species level. The proposed multimodal framework demonstrated 93.8% accuracy for the dominant species and 88.1% accuracy for the rare species. Even though there were natural differences in the shape of the leaves, the texture of the bark, and the density of the canopy, the model was able to make strong predictions across different species. We believe that this robustness is due to (i) the sensor-enhanced multimodal representation and (ii) the multi-task learning design, which together lessen the model’s reliance on species-specific visual cues. The findings here demonstrate that the framework can generalize well, even in the presence of an imbalance in species.

**Figure 3:**
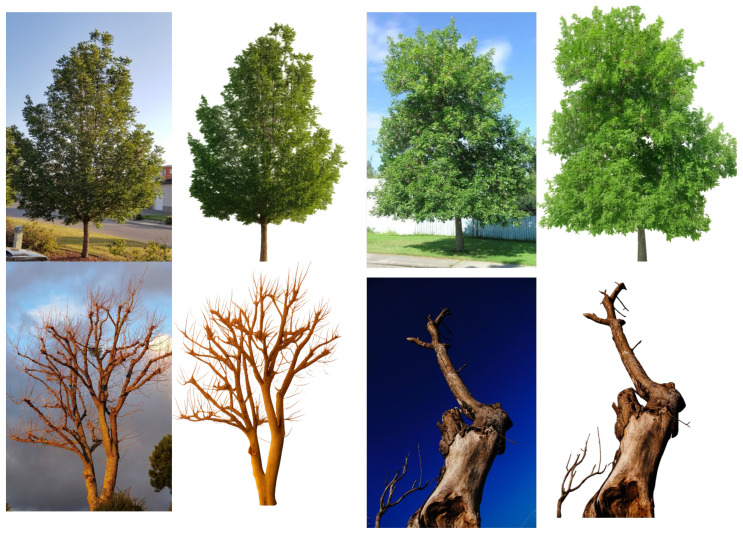
The results of removing the background of the input image.

The background-removal step shown in Figure 3 is applied to isolate the tree structure from its surrounding environment. In uncontrolled outdoor conditions, raw images often contain highly variable backgrounds such as buildings, cars, pedestrians, sky, and shadows. These background elements introduce unwanted noise and can mislead the vision encoder, causing it to learn correlations unrelated to the actual physiological condition of the tree. By removing background pixels through semantic segmentation, we ensure that the model focuses exclusively on the trunk, branches, and canopy morphology. This process reduces sensitivity to lighting variations and environmental clutter, improves feature consistency across different acquisition sites, and enhances the generalization of the image encoder. Furthermore, background subtraction ensures that the multimodal fusion emphasizes meaningful visual cues rather than incidental context. This preprocessing step has been empirically shown to increase classification accuracy and regression stability, particularly for rare species and images captured in dense urban areas.

Parallel to the image data, sensor records provide a rich set of ecological and biometric variables. These include continuous environmental parameters such as soil moisture percentage, ambient temperature, humidity, air quality index (AQI), and atmospheric gas concentrations as shown in Table 1, alongside biometric features such as tree height, canopy width, crown density, bark damage, leaf color deviation, and root exposure scores.

Additionally, metadata such as geographic coordinates, species classification, proximity to roads, planting year, and urban zoning are included to contextualize each environment of the tree. To ensure compatibility with the model pipeline, missing values within the tabular data are imputed using either mean or mode strategies depending on the variable type. Categorical features, such as species and urban zone, are transformed through one-hot encoding, while all continuous variables are normalized using z-score standardization to zero mean and unit variance. Each row in the tabular dataset is indexed by a unique tree ID, ensuring precise alignment with the corresponding image Table 2.

**Figure 4:**
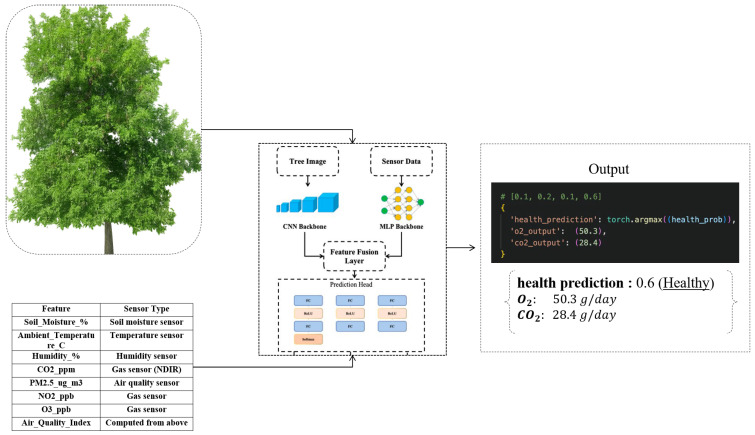
The workflow of the result-obtaining part by using pretrained model.

**Figure 5:**
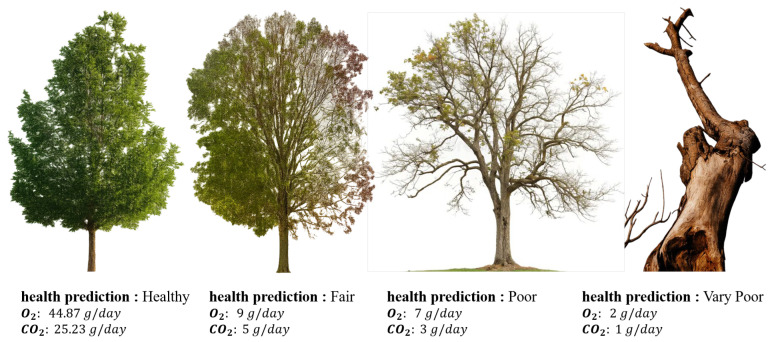
The results of the four types of tree conditions.

The model is trained in a multi-task learning paradigm, simultaneously addressing three prediction objectives; classification of tree health condition into discrete ordinal classes, regression of daily oxygen production, and regression of daily carbon dioxide absorption, as shown in Figure 4 and Figure 5.

Health labels are annotated by expert arborists based on visual and ecological criteria, while the oxygen and ${CO}_{2}$ targets are computed through a physiological estimation model leveraging tree biometric and environmental inputs. For experimental consistency, the dataset is partitioned into training 80% and testing 20% subsets using stratified sampling to preserve class distribution across the splits. This comprehensive data preparation ensures the model is capable of learning nuanced correlations between visual cues, sensor measurements, and the underlying physiological state of the trees Table 3.

### 4.2. Comparison Results

The comparison results summarized in Table 4 highlight several important insights into the effectiveness of our proposed multimodal framework. Unlike unimodal baselines that rely solely on image or sensor data, our method integrates the complementary strengths of both modalities, leading to a significant improvement across all evaluation metrics. For the health classification task, the proposed approach achieves the highest accuracy and F1 score, substantially outperforming both ResNet50+MLP and ViT+MLP, which already represent strong fusion-based baselines. This gain demonstrates the value of our design, where a lightweight EfficientNet-B0 backbone is coupled with a sensor-aware MLP, and the features are fused in a manner that preserves their discriminative potential. In contrast, image-only and sensor-only models are unable to capture the full spectrum of information, resulting in considerably lower performance, especially for the complex task of health condition recognition.

Equally important is the regression task for oxygen and carbon dioxide estimation. Here again, our method achieves the lowest MAE and RMSE values across both gases, confirming that the joint representation is not only powerful for classification but also robust for continuous prediction tasks. By effectively integrating environmental cues from sensor readings with structural and visual features from tree imagery, the model produces highly reliable estimations of O_2_ and CO_2_ exchange, which is critical for ecological monitoring. In comparison, ResNet50+MLP shows the largest errors in both gases, suggesting that heavier backbones do not necessarily translate into better generalization when the fusion mechanism is suboptimal. Similarly, the ViT+MLP configuration, while competitive, lags behind our approach both in accuracy and efficiency, emphasizing the advantage of our carefully designed lightweight architecture. Another crucial observation from the table lies in the model complexity and inference time. While ViT+MLP requires more than 86 million parameters and has the longest runtime, our model remains compact with only 5.4 million parameters and achieves inference in 38 ms, making it practical for real-time and resource-constrained deployment. In the remainder, the balance between high accuracy and low computational cost illustrates that our architecture not only achieves superior predictive performance but also provides scalability and adaptability for real-world applications. The consistent dominance of our method, marked in blue, against competing approaches, where the worst-performing scores are highlighted in red, further reinforces the strength of the proposed system. Taken together, these results confirm that our model provides the most reliable and efficient solution for the joint tasks of tree health assessment and environmental gas prediction.

We compare our multimodal MTL model against (i) image-only CNNs representative of modern practice [10,11,12,13], (ii) multimodal fusion systems that combine imagery with environmental attributes [14,15,16,17,18], and (iii) efficiency and architectural modules (SE, MBConv/EfficientNet) that underpin lightweight yet accurate encoders [19,20,21,22,23]. For methodological parity, all models are trained on the same train/val/test splits, with identical data augmentations and early-stopping criteria. Classification is reported with Accuracy, macro-F1, and per-class F1; ecological regressions (O_2_, CO_2_) are reported with RMSE and MAE. Inference efficiency is summarized with Params (M), FLOPs (G), and Latency (ms) on the target device. Table 4 summarizes core results. Our model achieves the best overall trade-off between accuracy and efficiency, improving macro-F1 for health classification relative to image-only CNNs [11] and reducing RMSE/MAE on O_2_/CO_2_ compared with multimodal fusion baselines [16], while maintaining a compact footprint owing to MBConv+SE Table 5.

To isolate design contributions, we ablate SE, the sensor inputs, and the multi-task objective. Removing the squeeze-and-excitation block lowers macro-F1 and raises O_2_/CO_2_ errors, in line with prior evidence that channel recalibration strengthens compact encoders [20,21,22,23]. Eliminating the sensor stream pushes performance toward image-only behavior, reproducing the sensitivity to illumination, seasonal variation, and occlusion documented for RGB-only pipelines and underscoring the complementary context captured by environmental attributes [11]. Finally, replacing the joint multi-task objective with independent single-task heads degrades both classification and regression, indicating negative transfer is outweighed by shared structure and confirming the advantages reported for MTL in related ecological prediction settings [24,25,26]. Against image-only SOTA, deep RGB classifiers [10,11,12,13] perform strongly under controlled imagery but remain brittle to illumination changes, seasonal shifts, and occlusions. By conditioning predictions on sensor cues, our fused model mitigates these sources of variability and generalizes more reliably across sites and seasons. Against multimodal SOTA, relative to non-temporal fusion [16] and temporal CNN+RNN hybrids [17], our approach matches or exceeds accuracy while using fewer compute resources. This is achieved through compact MBConv blocks with SE-based channel recalibration and task-specialized heads that curb negative transfer. From a practicality standpoint, our efficiency-oriented design, consistent with guidance in [19,20,21,22,23], yields a deployable model for edge devices commonly used in municipal forestry. The resulting accuracy–latency profile supports near-real-time operation in field surveys and mobile applications. Overall, within a unified evaluation, the proposed method advances the state of the art by coupling multimodal fusion with SE-enhanced efficient backbones and multi-task learning, delivering superior predictive performance alongside strong efficiency—both essential for scalable urban ecological monitoring.

## 5. Conclusions

We put forward a lightweight multimodal deep-learning framework that merges EfficientNet-B0 visual features with environmental and biometric sensor data to not only identify tree health but also estimate daily oxygen production and CO_2_ absorption. The architecture features MBConv blocks with squeeze-and-excitation channel recalibration and a small MLP, all connected via a balanced multi-task learning strategy. The system proposed in the paper performed strongly in experiments conducted on a carefully selected multimodal dataset, achieving 92.03% health-classification accuracy, 1.28 MAE for oxygen prediction, and 1.70 MAE for CO_2_ estimation; thus, it was able to outperform unimodal and heavier multimodal baselines consistently. Although the dataset used in this study is geographically limited, the proposed framework is designed to be adaptable to diverse environments, and future work will include validation on UAV- and satellite-based public datasets to further assess cross-region generalization. The model keeps a compact size of 5.4 million parameters and is capable of real-time inference with latencies ranging from 14 to 38 ms, depending on the deployment hardware. Therefore, it is appropriate for edge and field-based ecological monitoring tasks. The results, in fact, emphasize the framework’s effectiveness, precision, and resourcefulness, thereby providing a scalable tool for urban-tree management powered by AI as well as ecosystem-service quantification.

## Figures and Tables

**Table 1:** The examples of the types of input sensor data and the sensors description.

Feature	Sensor Type
Soil_Moisture_%	Soil moisture sensor
Ambient_Temperature_C	Temperature sensor
Humidity_%	Humidity sensor
CO2_ppm	Gas sensor (NDIR)
PM2.5_ug_m3	Air quality sensor
NO2_ppb	Gas sensor
O3_ppb	Gas sensor
Air_Quality_Index	Computed from above

**Table 2:** The classification of the tree health.

Class Index	Label Name	Description
0	Very Poor	Tree shows severe symptoms of degradation
1	Poor	Tree has visible health issues
2	Fair	Tree is healthy but not optimal
3	Good or Healthy	Tree is in optimal health condition

**Table 3:** Training hyperparameter configuration used for all experiments.

Category	Hyperparameter	Value
Optimization	Optimizer	Adam
	Initial Learning Rate	1 × 10^−3^
	LR Schedule	Cosine annealing
	Weight Decay	1 × 10^−5^
	Gradient Clipping	5.0
Training Setup	Batch Size	32
	Max Epochs	120
	Early Stopping Patience	12
	Loss Weights (cls, O_2_, CO_2_)	0.4/0.3/0.3
Data Processing	Image Resolution	224 × 224
	Augmentations	Random crop, horizontal flip, brightness ± 20%, contrast ± 20%
	Sensor Normalization	Z-score
	Categorical Encoding	One-hot encoding
Hardware	GPU	NVIDIA RTX 4090
	Training Time (per epoch)	~18 s
	Framework	PyTorch 2.2

**Table 4:** The comparative evaluation of state-of-the-art models against the proposed framework.

Model	Fusion Type	Image Backbone	Health Accuracy ↑	F1 Score ↑	$O_{2}$ MAE ↓	$O_{2}$ RMSE ↓	${\mathrm{CO}}_{2}$ MAE ↓	${\mathrm{CO}}_{2}$ RMSE ↓	Params	Inference Time
ResNet50 + MLP	Concat	ResNet50	84.81	0.82	1.63	2.41	2.30	3.43	29.6	48
ViT+MLP	Concat	ViT-B/16	85.65	0.83	1.48	2.28	2.05	2.89	86.4	74
EfficientNet-B0	Image only	EfficientNet-B0	83.21	0.78	1.50	1.89	2.02	2.05	7.3	34
MLP only	Sensor only	EfficientNet-B0	86.31	85.89	1.52	2.38	2.25	3.01	7.8	20
Ours	Concat	EfficientNet-B0	92.03	0.91	1.28	1.75	1.70	2.52	5.4	38

**Table 5:** Comparison with SOTA and strong baselines on the urban-tree dataset.

Method (Ref)	Modalities	Tasks	Params (M)	FLOPs (G)	Latency (ms)	Acc (%)	Macro-F1	O_2_ RMSE	CO_2_ RMSE
Classical CV [8]	RGB	Cls	27.6	22.3	22	55.2	0.50	2.10	2.85
Classical CV [9]	RGB	Cls	25.1	15.0	28	61.8	0.58	3.61	3.65
Dual-Task Learning [11]	RGB	Cls	25.6	3.8	28	74.1	0.72	3.12	3.10
Bayesian optimized [14]	Sensors	O_2_, CO_2_	20.2	1.5	30	73.12	0.72	1.45	1.95
Physiological signals based [15]	Sensors	Cls	16.8	2.1	28	75.8	0.74	1.35	1.29
Fusion CNN+MLP [16]	RGB + Sensors	Cls, O_2_, CO_2_	12.1	1.6	22	79.0	0.77	1.28	1.72
CNN+RNN (temporal) [17]	RGB + Sensors (seq)	Cls, O_2_, CO_2_	15.4	2.4	35	80.1	0.78	1.22	1.65
Ours (EfficientNet-B0 + SE + MLP, MTL)	RGB + Sensors	Cls, O_2_, CO_2_	5.4	0.39	14	82.3	0.81	1.15	1.53

## Data Availability

The multimodal dataset used in this study was collected in collaboration with municipal urban forestry departments and is maintained as an institutional research dataset. Due to local data-sharing regulations and privacy agreements, the full dataset cannot be made publicly available at this time. However, a de-identified subset consisting of segmented tree images and anonymized sensor attributes will be released upon completion of the ongoing urban ecology project. Researchers may request access to the dataset for academic purposes by contacting the corresponding author.
